# Directed Use of the Internet for Health Information by Patients With Chronic Kidney Disease: Prospective Cohort Study

**DOI:** 10.2196/jmir.2848

**Published:** 2013-11-15

**Authors:** Clarissa Jonas Diamantidis, Wanda Fink, Shiming Yang, Marni R Zuckerman, Jennifer Ginsberg, Peter Hu, Yan Xiao, Jeffrey C Fink

**Affiliations:** ^1^Department of MedicineDivision of NephrologyUniversity of Maryland School of MedicineBaltimore, MDUnited States; ^2^Department of MedicineDivision of NephrologyVeterans Affairs Maryland Health Care SystemBaltimore, MDUnited States; ^3^Department of Computer ScienceUniversity of Maryland Baltimore CountyBaltimore, MDUnited States; ^4^Department of AnesthesiologyR Adams Cowley Shock Trauma CenterUniversity of MarylandBaltimore, MDUnited States; ^5^Baylor Health Care Quality Institute for Health Care Research and ImprovementOffice of Patient SafetyBaylor Health Care SystemDallas, TXUnited States; ^6^Department of Epidemiology and Preventive MedicineSchool of MedicineUniversity of MarylandBaltimore, MDUnited States

**Keywords:** chronic kidney disease, health information technology, patient safety

## Abstract

**Background:**

Health information technology has become common in the care of patients with chronic diseases; however, there are few such applications employed in kidney disease.

**Objective:**

The aim of the study was to evaluate the use of a website providing disease-specific safety information by patients with predialysis chronic kidney disease.

**Methods:**

As part of the Safe Kidney Care (SKC) study, an educational website was designed to provide information on safety concerns in chronic kidney disease. Phase I study participants were provided a medical alert accessory with a unique ID number, the Safe Kidney Care website, and an in-person tutorial on the use of the Internet and accessing the SKC website at baseline. Participants were asked to visit the website and enter their unique ID as frequently as they desired over the next 365 days or until their annual follow-up visit, whichever occurred first. Participants’ visits and dwell times on specific safety modules were tracked using embedded webpage PHP scripts linked to a MySQL database, enabling the collection of website usage statistics.

**Results:**

Of 108 Phase I participants, 28.7% (31/108) visited the website from 1-6 times during the observation period (median follow-up 365 days). Median access time was 7 minutes per visit (range <1-46) and 13 minutes per person (range <1-123). The three most frequently visited pages were “Renal function calculator”, “Pills to avoid”, and “Foods to avoid”. High school education and frequent Internet use were significantly associated with website entry (*P*=.02 and *P*=.03, respectively).

**Conclusions:**

Preliminary results show general interest in a Web-based platform designed to improve patient safety in chronic kidney disease.

**Trial Registration:**

Clinicaltrials.gov NCT01407367; http://clinicaltrials.gov/show/NCT01407367 (Archived by WebCite at http://www.webcitation.org/6KvxFKA6M).

## Introduction

Health information technology is expected to play a growing role in medical care delivery over the coming years [1]. The Internet has become a major source of educational health materials for both patients and providers and mobile health applications are being developed for a wide variety of diseases and health conditions [2-7]. For health IT applications to have a beneficial impact on disease outcomes, they must be designed to match the predominant technologic proficiency (e-literacy) of the target population. While the use of computers, the Internet, and mobile devices continues to rise in the United States [8,9], many chronic disease populations are not the target of commercial IT developers. The population with chronic kidney disease is one such population, as it includes a high preponderance of individuals who are older, of lower socioeconomic status, and with lower health literacy [10,11]. Therefore, it is unknown whether health IT tools intended for chronic kidney disease can be effective.

The Safe Kidney Care (SKC) study was a prospective cohort designed to gauge adverse safety events in chronic kidney disease. In Phase I of this study, we set out to evaluate the acceptance and initial use of a health IT system including a device designed to increase recognition of chronic kidney disease (medical alert accessory) and linked to a website. The medical alert bracelet was devised for the purpose of alerting patients and providers of a patient’s diagnosis of chronic kidney disease and directing these individuals to a website informing patients, family members, and providers about the unique patient safety concerns associated with chronic kidney disease management. We tracked the incidence of study participants’ initial entry into the website over a one-year period and their prioritization of chronic kidney disease patient safety concerns.

## Methods

### Study Population

The SKC cohort study (Clinicaltrials.gov NCT 01407367) was approved by the University of Maryland School of Medicine Institutional Review Board and Veterans Affairs Maryland Health Care System Baltimore Research and Development committee. The primary objective of the SKC cohort study was to examine the relationship between chronic kidney disease recognition and patient safety. The objective of Phase I of the SKC cohort was to evaluate a medical alert accessory (American Medical ID, Houston, TX) noting the participant’s diagnosis of chronic kidney disease and directing patients and providers to the Safe Kidney Care website offering information on common patient safety concerns pertinent to chronic kidney disease. To be eligible for the SKC cohort, participants needed two measures of renal function with an estimated glomerular filtration rate (eGFR) of less than 60 ml/min/1.73 m^2^ at least 90 days apart and no more than 18 months prior to enrollment. Participants were excluded for Phase I if they were expected to reach end-stage renal disease or die within one year from enrollment or if they had skin sensitivity to silver or stainless steel. Primary sources for recruitment and enrollment were the University of Maryland Medical System and Baltimore Veterans Affairs Medical Center Early Renal Insufficiency disease management clinics.

### Safe Kidney Care (SKC) Cohort Study Procedures

**Figure 1:**
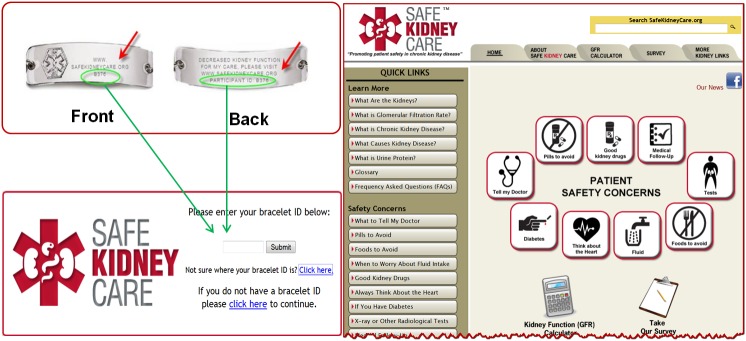
Medical alert accessory and log-in page of Safe Kidney Care website patient portal.

Upon enrollment into the SKC cohort, participants were seen at baseline and then annually in-center with a 6-month interim telephone follow-up visit. At the baseline visit, Phase I participants were provided with a fitted medical alert accessory of their choosing (bracelet or necklace) engraved with the message: “Decreased kidney function”. For safe care, please visit the Safe Kidney Care website and a unique study number. This unique ID number did not contain any personally identifiable information. After baseline data collection and provision of the medical alert accessory, the study coordinator provided participants with a demonstration and short tutorial on accessing the Internet and SKC website, where to enter their unique ID number, and a tour of the website and its features. At completion of the tutorial, participants were encouraged to use the website, along with family members and providers. They were instructed to enter their unique ID number when accessing the website’s patient portal so that their usage could be tracked, although this was not required for entrance into the website (Figure 1).

### Safe Kidney Care Website Development

Prior to the commencement of enrollment into the SKC cohort, an extensive literature review was conducted to identify chronic kidney disease-pertinent patient safety concerns. Approximately 800 academic and community nephrologists were then invited to participate in a survey to prioritize identified candidate patient safety concerns and to suggest additional potential patient safety events not previously considered. The feedback by participating respondents (n=142) was then used as the basis for development of the SKC website, which covers the most commonly identified chronic kidney disease-specific patient safety concerns along with other general nephrology educational topics. The resulting modules were reviewed by an expert panel convened for the SKC project with additional modifications made based on their input. The website contains both a patient and family member portal, which provides module descriptions in lay terms, and a health care provider portal, which uses common medical terminology.

The resultant SKC website was designed using guidelines for color and content layout incorporated from the 508 Compliance and Disabilities Act and heuristic design [12,13]. Emphasis was placed on ease of readability, with text written to target a 6^th^ grade reading level in the patient portal. The primary placement of the “Safety Concerns” was in a circular distribution to avoid the implication of prioritization of topics. Images were designed to be static because of the anticipated limited access to Adobe Flash (or other multimedia platform). The website was designed to limit the need to scroll; however, on pages with a requirement for scrolling, the most important content was placed above the fold so as to ensure primary focus on the safety content. The capability to increase text size on every page was installed and displayed and meaningful, relatable icons were chosen for easy recognition of content topics (eg, picture of a stethoscope to represent “What to Tell My Doctor”). A quick-launch side panel, entitled “Learn More”, included links to pages on the SKC website which provide users with basic information about the function of the kidneys, what is meant by kidney disease, definition of the glomerular filtration rate (GFR), and a glossary of commonly used medication terms (eg, “blood pressure”, “dialysis”, and “nephrologist”). The website markup and content were constructed using HTML with embedded functions programmed using PHP programming language (Version 5.3.5). The design was conceived with the goal of optimal use on all Internet portals. Usability testing was conducted on a small representative sample and previously reported [14]. Modifications to the website were instituted based on feedback from usability participants.

### Safe Kidney Care Website Surveillance

To evaluate the receipt of information designed for the targeted audience, we tracked activities on the SKC website of each bracelet ID assigned to study group participants, including page viewing frequency, browsing time for each page, and feedback. Due to flexibility and security concerns, our implemented computer routine was believed to surpass other available online tracking and analysis services (eg, Google Analytics), as our own implementation and hosting permitted precise tracking defined for the study purposes with data stored securely on our local server.

The website was programmed to track both registered and anonymous users through PHP scripts embedded in each webpage. The PHP scripts were linked with a database built on the MySQL server, to collect and store predefined website usage statistics. For each distinct participant log-in or anonymous guest visit, a unique session number was assigned to label all actions during the visit until the session expired or the visitor started a new session. Medical alert accessory unique ID, session number, log-in time, and IP address were recorded into a table for each independent visit. During a session, each click of an internal or external link was captured and a unique record was generated, including session number, page/link entry time, and page/link ID. With this information, it was possible to recreate the visiting pathway for each visit, to count total pages read in each session, and to calculate other pertinent statistics such as frequency of each page visited in each session, or number of visits from a registered user. It is generally difficult to determine the time when a visitor leaves a page because the visitor may leave the computer for an interruption and return after some time; hence, it was not possible to ascertain precisely how long a visitor focused on the content of the webpages. Nevertheless, the dwelling time for each page was estimated, under the assumption that a visitor would not spend an extended time on a single page but would continue to browse different pages. At the same time, a minimum length of time must also be spent on each page to conduct effective reading. Therefore, an effective dwelling length on a page was counted if the time difference between two distinct webpage entry times (within the same session) was longer than 5 seconds and less than 10 minutes.

### Outcomes

The primary outcome in this analysis was the first entry into the SKC website with input of a unique ID number by SKC study participants at any time following completion of their baseline SKC visit until their first annual follow-up visit or 365 days of observation, whichever occurred first. Access of the health care provider portal was not analyzed for the purpose of this study. The number of days from baseline SKC visit was recorded, as was the length of dwell time on the website and on selected modules. Modules were ranked by frequency of selection if selected at least once during the participant’s initial visit on the website.

### Covariates

Additional factors measured at baseline SKC visit and included in the analysis were demographic characteristics, socioeconomic status, education, health literacy and numeracy [15], and ease and frequency of use of the Internet. Baseline serum creatinine was measured and used to estimate the GFR with the abbreviated Modified Diet in Renal Disease equation [16].

### Statistical Methods

For descriptive analyses, continuous variables were presented with mean and standard error (SE) and Student’s *t* test for comparison across groups. Binomial and categorical variables were expressed as N (%) with comparisons made with the chi-square test. Stepwise logistic regression was used to examine factors that predict incident registration into the SKC website. All factors were used in a forward stepwise inclusion procedure with retention of only those factors with significance of *P*<.05.

## Results

Enrollment commenced for Phase I of the SKC cohort on April 15, 2011 and the last of 108 participants completed the baseline visit on January 23, 2012. A medical alert bracelet was selected by 55 (50.9%, 55/108) of participants with 53 (49.1%, 53/108) choosing the medical alert necklace. The median observation time was 365 days (range 305-365). Table 1 shows the demographic and baseline characteristics of all participants. Mean age of SKC participants was 64 years (SD 11) with a mean estimated GFR of 42 ml/min/1.73m^2^ (SD 14). Of note, 10 enrolled participants were estimated to have eGFRs >60 ml/min/1.73^2^ at their baseline visit, despite two screening eGFRs that fit enrollment criteria of eGFR <60 ml/min/1.73m^2^. The majority of participants were black, unmarried, with a household income of ≤ US$50,000 annually (72.2%, 78/108; 55.6%, 60/108; and 68.5%, 74/108, respectively). While 86.1% of participants (93/108) reported availability of computer access, 45.4% (49/108) estimated their Internet usage to be less frequent than weekly or daily.

**Figure 2:**
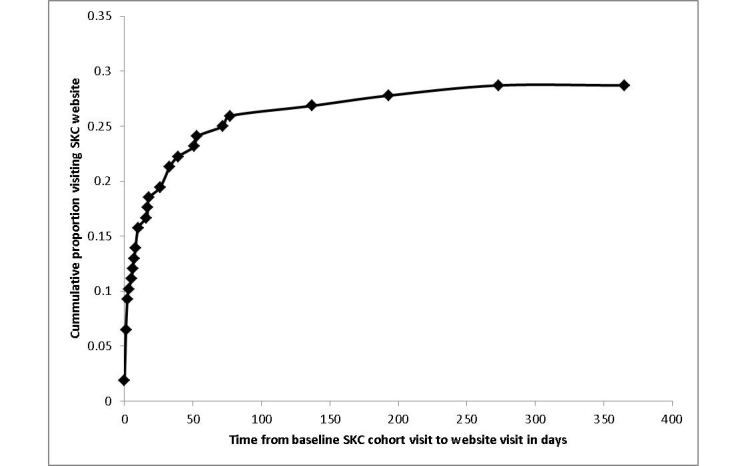
Cumulative incidence of Safe Kidney Care website log-in.

Figure 2 shows the results of the SKC website usage at the end of observation for all participants. To date, 28.7% (31/108) of participants visited the website since study enrollment a median of 1 time (range 1-6). Median SKC website access time per visit was 7 minutes (range <1-46). Median cumulative SKC website access time per person was 13 minutes (range <1-123) with a cumulative access time by all 31 participants of 669 minutes. The three most frequently visited pages were “Renal function calculator”, “Pills to avoid”, and “Foods to avoid” (Table 2).

The adjusted proportion of individuals with SKC website log-in by baseline characteristic is shown in Table 3 **.** After stepwise logistic analysis, pertinent and significant factors predictive of incident use of the SKC website were high school diploma (OR 3.22, 95% CI 1.18-8.81) and frequent use of the Internet (OR 3.31, 95% CI 1.13-9.72), *P*=.02 and *P*=.02, respectively.

**Table 1:** Safe Kidney Care participants’ baseline demographics.

Baseline characteristic		n (%)
**Age**		
	≤50	10 (9)
	51-64	46 (43)
	65+	52 (48)
**Gender**		
	Male	70 (65)
	Female	38 (35)
**Race/ethnicity**		
	Black	78 (72)
	Non-black	30 (28)
**Annual household income** ^a^		
	≤$50,000	74 (69)
	>$50,000	34 (31)
**Education**		
	≤High school diploma	51 (47)
	>High school diploma	57 (53)
**Marital status**		
	Not currently married	60 (56)
	Currently married	48 (44)
**Employment status**		
	Not employed full-time	96 (89)
	Employed full-time	12 (11)
**Baseline estimated glomerular filtration rate (mL/min/1.73m** ^**2**^ **)** ^b^		
	Stage 2 (≥60)	10 (9)
	Stage 3a (45-59)	33 (31)
	Stage 3b (30-44)	47 (44)
	Stage 4 or 5 (<30)	18 (17)
**Health literacy** ^c^		
	Marginal/inadequate (TOFHLA<67)	32 (30)
	Adequate (TOFHLA≥67)	76 (70)
**Computer access available**		
	No	15 (14)
	Yes	93 (86)
**Internet use**		
	Rarely/never	49 (45)
	Often	59 (55)

^a^Five participants declined to answer income status.

^b^Ten participants had baseline estimated glomerular filtration rates (eGFR) ≥60 mL/min/1.73m^2^ despite qualifying screening eGFRs of <60 mL/min/1.73m^2^.

^c^Seven participants were unable to complete the abbreviated Test of Functional Health Literacy Assessment (TOFHLA) due to conditions such as blindness.

**Table 2:** Most frequently visited SKC webpages.

Webpage title	Total visits	Cumulative time (minutes)	Average time per visit (minutes)
Kidney Function (GFR) calculator	36	50	1
Pills to avoid	34	55	2
Foods to avoid	25	64	3
What to keep in mind about the “good” kidney drugs	17	58	3
When you need a special type of x-ray or other radiologic test…	17	28	2
What is glomerular filtration rate?	16	34	2
When to worry about your fluid intake	12	37	3
What is urine protein?	12	20	2
Frequently asked questions	10	33	3
Take our survey	10	28	3
What is chronic kidney disease (CKD) or weak kidneys?	10	26	3
If you have diabetes…	10	21	2
About the Safe Kidney Care website	10	19	2
What to tell my doctor, nurse, or pharmacist	10	17	2

**Table 3:** Predictors of Safe Kidney Care website log-in.

Baseline characteristic		Unadjusted proportion	Adjusted proportion (95% CI)
**Age**			
	≤50	30	Reference (ref)
	51-64	28	47 (13-84)
	65+	29	47 (13-84)
**Gender**			
	Male	27	ref
	Female	32	43 (20-69)
**Race/ethnicity**			
	Black	24	ref
	Non-black	40	31 (11-61)
**Annual household income** ^a^			
	≤$50,000	20	ref
	>$50,000	47	40 (18-68)
**Education**			
	≤High school diploma	14	ref
	>High school diploma	42	28 (11-55)
**Marital Status**			
	Not currently married	23	ref
	Currently married	35	20 (8-44)
**Employment status**			
	Not employed full-time	26	ref
	Employed full-time	50	28 (8-65)
**Baseline estimated glomerular filtration rate (mL/min/1.73m** ^**2**^ **)** ^b^			
	Stage 2 (≥60)	30	ref
	Stage 3a (45-59)	33	31 (7-72)
	Stage 3b (30-44)	32	27 (6-68)
	Stage 4 or 5 (<30)	11	9 (1-48)
**Health literacy** ^c^			
	Marginal/inadequate (TOFHLA<67)	22	ref
	Adequate (TOFHLA≥67)	32	13 (4-36)
**Computer access available**			
	No	7	ref
	Yes	32	9 (1-52)
**Internet use**			
	Rarely/never	12	ref
	Often	43	36 (12-68)

^a^Five participants declined to answer income status.

^b^Ten participants had baseline estimated glomerular filtration rates (eGFR) ≥60 mL/min/1.73m^2^ despite qualifying screening eGFRs of <60 mL/min/1.73m^2^.

^c^Seven participants were unable to complete the abbreviated Test of Functional Health Literacy Assessment (TOFHLA) due to conditions such as blindness.

## Discussion

### Principal Findings

In this study, we have described the longitudinal access and usage of a Web-based reference on the unique concerns of patient safety in chronic kidney disease based on the provision of a medical alert accessory with a unique identifier and prompt for website use. The study evaluates the alignment of a commonly used medical alert device, designed to enhance awareness of chronic kidney disease, with a health IT portal offering disease-pertinent information to the target population. The results offer an assessment on the likelihood that individuals with chronic kidney disease and their family members will utilize this alert system intended to increase disease awareness and improve patient safety. The findings suggest a substantial proportion of the SKC Phase I sample, designed to be representative of the chronic kidney disease population, were capable and motivated to access the website to view the developed patient safety modules. The results reveal the population’s prioritization of safety concerns specific to chronic kidney disease. Moreover, the report on dwell time provides indication of the importance users placed on the content.

Several reports show that patients with chronic disease who are empowered with IT tools for monitoring, training, and self-management have improved outcomes [2-7], yet few prior investigations evaluate the usage of these applications in general or those specifically designed for the chronic kidney disease population. While online educational materials designed to promote patient education and community awareness of kidney disease are available via high-quality programs such as the National Kidney Disease Education Program [17] and the National Kidney Foundation [18], the use of these materials by the target population remains unknown. Our study is unique in its demonstration of the feasibility of acceptance and utilization of a hybrid system combining a longstanding physical device (a variant of which has been most commonly utilized in kidney disease to preserve potential vascular access sites) [19] with its value enhanced by a usability-tested health IT portal [14] providing extensive information relevant to the chronic kidney disease population.

Introduction of online tools does not guarantee their usage by the target population and the results from this study support prior examinations that estimate use of online tools to be highly variable. Our findings align with studies in other chronic diseases, which reveal inconsistency in website use, but self-reported or measured use has been noted to range from approximately 10-40% of those queried [20-22]. Adults in the United States living with chronic disease are significantly less likely than healthy adults to have access to the Internet (62% vs 81%), but once online, those with chronic disease are more likely to use social media and online tools to share information and obtain support from their peers [23]. Individuals with chronic disease still report an overwhelming preference to receive health-related education and advice from a professional source such as a health care provider [24], which emphasizes the role of the Internet as an accessory to, rather than a replacement for, quality provider-patient interactions. The results presented here suggest information needs in the areas most frequently visited on the website, even for face-to-face encounters.

### Limitations

The study presented has limitations related to the descriptive nature of the results, which may limit generalizability. While approximately 30% of participants visited the SKC website, 70% did not, which is an important finding. These individuals who did not access the website were not specifically asked to comment on reasons for their lack of participation (eg, lack of interest rather than access), which may not be captured in the demographic evaluation of computer and Internet access, but likely is highly relevant to future work using health IT applications in chronic kidney disease. The use of nephrologists only in identifying specific areas of safety concerns in chronic kidney disease was limited in that it did not include advanced practitioners or primary care providers, who play important roles in caring for individuals with chronic kidney disease and undoubtedly would have added value to the content. Further, we did not incorporate patients in the initial design of the website; however, usability testing was performed with such a population using an iterative process used to finalize the SKC website [14]. Finally, the relatively short follow-up of participants does not allow us to yet comment on the impact of the conjoined alert accessory and website resource on patient safety outcomes in chronic kidney disease.

### Conclusions

Patient safety is a significant problem in kidney disease and online education tools such as the SKC website may serve as platform to educate individuals with chronic kidney disease about potential hazards related to their condition. Further examination is needed to assess individuals’ long-term utilization and dissemination of such materials and the impact of such resources in preventing safety events in chronic kidney disease.

## Abbreviations

eGFR: estimated glomerular filtration rate
SKC: Safe Kidney Care
TOFHLA: Test of Functional Health Literacy Assessment

## References

[1] Goodson JD (2010). Patient Protection and Affordable Care Act: promise and peril for primary care. Ann Intern Med.

[2] Gruman JC (2011). Making health information technology sing for people with chronic conditions. Am J Prev Med.

[3] Cummings E, Turner P (2009). Patient self-management and chronic illness: evaluating outcomes and impacts of information technology. Stud Health Technol Inform.

[4] Eland-de Kok P, van Os-Medendorp H, Vergouwe-Meijer A, Bruijnzeel-Koomen C, Ros W (2011). A systematic review of the effects of e-health on chronically ill patients. J Clin Nurs.

[5] Morren M, van Dulmen S, Ouwerkerk J, Bensing J (2009). Compliance with momentary pain measurement using electronic diaries: a systematic review. Eur J Pain.

[6] Krishna S, Boren SA, Balas EA (2009). Healthcare via cell phones: a systematic review. Telemed J E Health.

[7] Bennett GG, Warner ET, Glasgow RE, Askew S, Goldman J, Ritzwoller DP, Emmons KM, Rosner BA, Colditz GA, Be Fit‚ Be Well Study Investigators (2012). Obesity treatment for socioeconomically disadvantaged patients in primary care practice. Arch Intern Med.

[8] Smith, A.

[9] Purcell, K.

[10] U.S. Renal Data System Bethesda, MD: National Institute of Diabetes and Digestive and Kidney Disease, National Institute of Health.

[11] Devraj R, Gordon EJ (2009). Health literacy and kidney disease: toward a new line of research. Am J Kidney Dis.

[12] Website Design 508 Compliance.

[13] Cyr DH, Head M, Larios H (2010). Colour appeal in website design within and across cultures: A multi-method evaluation. International Journal of Human-Computer Studies.

[14] Diamantidis CJ, Zuckerman M, Fink W, Hu P, Yang S, Fink JC (2012). Usability of a CKD educational website targeted to patients and their family members. Clin J Am Soc Nephrol.

[15] Baker DW, Williams MV, Parker RM, Gazmararian JA, Nurss J (1999). Development of a brief test to measure functional health literacy. Patient Educ Couns.

[16] Levey AS, Bosch JP, Lewis JB, Greene T, Rogers N, Roth D (1999). A more accurate method to estimate glomerular filtration rate from serum creatinine: a new prediction equation. Modification of Diet in Renal Disease Study Group. Ann Intern Med.

[17] National Kidney Disease Education Program (NKDEP): Improving the understanding, detection, and management of kidney disease.

[18] National Kidney Foundation TM.

[19] Vachharajani T (2009). Medical alert bracelet: an effective way to preserve veins for future dialysis vascular access in patients with chronic kidney disease. Medscape J Med.

[20] Beckjord EB, Finney Rutten LJ, Squiers L, Arora NK, Volckmann L, Moser RP, Hesse BW (2007). Use of the internet to communicate with health care providers in the United States: estimates from the 2003 and 2005 Health Information National Trends Surveys (HINTS). J Med Internet Res.

[21] Fleisher L, Kandadai V, Keenan E, Miller SM, Devarajan K, Ruth KJ, Rodoletz M, Bieber EJ, Weinberg DS (2012). Build it, and will they come? Unexpected findings from a study on a Web-based intervention to improve colorectal cancer screening. J Health Commun.

[22] Zhang Y, Jones B, Spalding M, Young R, Ragain M (2009). Use of the internet for health information among primary care patients in rural West Texas. South Med J.

[23] Fox, S.

[24] Kuehn BM (2011). Patients go online seeking support, practical advice on health conditions. JAMA.

[25] American Medical ID.

